# Proposed Implementation of a Patient-Centered Self-Assessment Tool for Patients with Neuroendocrine Tumors among Academic and Community Practice Sites: The City of Hope Model

**DOI:** 10.3390/jcm12031229

**Published:** 2023-02-03

**Authors:** Christiana Joy Crook, Lisa Yen, Kathleen Ta, Misagh Karimi, Danny Nguyen, Richard T. Lee, Daneng Li

**Affiliations:** 1 Department of Medical Oncology & Therapeutics Research, City of Hope National Medical Center, Duarte, CA 91010, USA; 2 Learn Advocate Connect Neuroendocrine Tumor Society, Denver, CO 80237, USA; 3 Department of Medical Oncology & Therapeutics Research, City of Hope Newport Beach Fashion Island, Newport Beach, CA 92660, USA; 4 Department of Medical Oncology & Therapeutics Research, City of Hope Irvine Sand Canyon, Irvine, CA 92618, USA; 5 Department of Supportive & Integrative Medicine, City of Hope Orange County Lennar Foundation Cancer Center, Irvine, CA 92618, USA

**Keywords:** neuroendocrine tumors, self-assessment, NET VITALS, implementation science

## Abstract

Neuroendocrine tumors are a rare type of cancer found in hormone-producing cells throughout the body. Research on disease-specific patient education assessments in this population is lacking. We previously demonstrated the feasibility and validity of NET VITALS, a patient-centered self-assessment designed to improve patients’ knowledge of their neuroendocrine tumor diagnosis/treatment and facilitate communication with their physician. In this report, we provide a brief overview of patient assessments that have been used for patients with neuroendocrine tumors. We summarize NET VITALS and present a proposed infrastructure for its implementation into standard clinical care in both academic and community practice settings at City of Hope. Incorporating NET VITALS into standard of care treatment for patients with neuroendocrine tumors may improve patients’ overall clinical care experience.

## 1. Introduction

Neuroendocrine tumors (NETs) are hormone-producing tumors that develop from endocrine cells throughout the body [1]. Although the incidence of NETs is rising, NETs are a rare diagnosis, with 8.3 cases per 100,000 individuals diagnosed in the United States in 2018 [2,3]. Given that NETs often present with nonspecific symptoms, such as diarrhea, bloating, abdominal cramping, and flushing [4,5], delays in diagnosis are common, with patients reporting a median of 9.2 years between the development of symptoms and final diagnosis [6]. As a result, patients often present with advanced or metastatic disease at diagnosis [7,8].

The relatively small number of patients with NETs may be a contributing factor to the lack of research regarding education and treatment experiences of this patient population. Patients with NETs often report poor clinical experiences, with many patients expressing frustration with the lack of information provided and poor communication with their treating physician [7,8,9]. Educational tools for patients with breast, prostate, and liver cancers have demonstrated improvements in patient understanding and satisfaction with the information received from their treating physician [10,11]. These positive results suggest that patients with NETs could also benefit from patient-centric educational tools.

In this report, we provide a brief overview of patient-centered self-assessments, with an emphasis on tools specifically designed for patients with NETs. We describe NET VITALS, a patient-centered self-assessment tool created by NET patient advocates and physicians, and propose a strategy for its implementation at City of Hope. Our goal is to establish a robust clinical infrastructure for the implementation of NET VITALS that could improve the treatment experience of patients with NETs and contribute to an increase in patients’ overall well-being.

## 2. Patient-Centered Assessments

Patient self-assessments have been developed for a variety of situations. Here, we describe disease-agnostic and disease-specific self-assessments that have been used among patients with NETs.

### 2.1. Disease-Agnostic Patient Assessments: Quality of Life

Many disease-agnostic patient self-assessments are designed to assess quality of life. A well-known example is the European Organization for Research and Treatment of Cancer (EORTC) Quality of Life Questionnaire-Core 30 (QLQ-C30) [12]. This 30-item questionnaire has been used among patients with a variety of diagnoses [13,14,15]. Other examples of patient-centered quality of life tools include the Health Outcomes Tool, Attitude Scale, Now vs Later tool, Prognosis and Treatment Perceptions Questionnaire, and Short Form 36 health survey questionnaire [16,17,18,19,20,21]. These tools allow patients to clearly identify treatment goals and current health status and can provide physicians with clarity regarding patients’ preferences, which can potentially improve patient–physician communication. Moreover, these tools have successfully been used in NET patient populations in the contexts of clinical trials and observational studies, emphasizing their utility [22,23,24,25,26,27,28,29]. However, patients’ knowledge of their disease and treatment is not analyzed with these tools, leaving a gap in patient education. While these tools can increase patient awareness and potentially improve patient–physician communication, they lack disease-specific questions that may provide greater insight for both the patient and physician.

### 2.2. NET-Specific Patient Assessments

Although there are numerous examples of disease-specific patient educational tools and assessments [10,11,30], there are few examples of assessments designed specifically for patients with NETs. The International Neuroendocrine Cancer Alliance (INCA) conducted a global survey of patients with NETs in 2014 that investigated patients’ knowledge of their disease and their perspective on disease burden and treatment experience [7,8,31]. While this anonymous survey provided the NET research community with additional details regarding patient perspectives on their NET disease burden, it did not allow for any follow-up with patients, nor did it provide patients with a way to improve communication with their physician. In 2017, INCA conducted another international survey of patients/families, healthcare providers, and patient advocates and reported that patients with NETs continue to struggle with a lack of information [32]. Only 30% of patients stated that they were provided with sufficient information from their healthcare provider at diagnosis. However, 59% of healthcare providers surveyed believed that they provided patients with sufficient information, highlighting a lack of communication between patients with NETs and their healthcare providers.

The EORTC created a quality of life questionnaire for patients with gastrointestinal NETs (EORTC QLQ-GINET21); this 21-item questionnaire has been validated in patients with liver, pancreatic, and other gastrointestinal NETs [33,34]. This questionnaire is often used in conjunction with the EORTC QLQ-C30 to obtain a more comprehensive picture of a NET patient’s health-related quality of life [23,26,27,35]. While the EORTC QLQ-GINET21 has allowed patients with gastrointestinal NETs to provide their physicians with information about their current quality of life, some of the questions may not be relevant for patients with non-functional tumors, and patients with non-gastrointestinal NETs are not included in the target patient population. Spolverato et al. designed a quality of life questionnaire specifically for patients with NET liver metastases that incorporated elements from the EORTC QLQ-C30, EORTC QLQ-GINET21, and Norfolk Quality of Life tool for NETs [36,37]. While this questionnaire is useful for this patient population, it does not assess patient knowledge or perception of information received.

To address the issue of patient satisfaction with information received, Bouma et al. developed a web-based information system designed to improve patient satisfaction with the amount and quality of information they were able to access about their diagnosis [38]. Although an initial feasibility study suggested that patients experienced an improvement in quality of life and were satisfied with the application, a randomized controlled trial comparing the web-based application to standard of care treatment found no difference in perception of information received or satisfaction with information received [39]. However, a 26-week multidisciplinary educational intervention for patients recently diagnosed with NETs reported improvements in patients’ general self-efficacy and health-related quality of life [28], suggesting that educational interventions in this patient population require further optimization to maximize their benefit to patients.

The NET Cancer Health Storylines mobile application was developed to allow patients with NETs to track the frequency and severity of their symptoms and monitor additional health outcomes such as nutritional concerns, medications, and sleep [40]. Adams et al. investigated the health-related quality of life of patients using the application who were receiving somatostatin analog treatment; they observed a decrease in reported physical symptoms on the EORTC QLQ-C30 and EORTC QLQ-GINET21 over time, suggesting that the act of tracking symptoms may improve patients’ perception of changes in symptoms [41].

In terms of treatment planning, Wagner et al. designed a multicriteria decision analysis framework for NET patients and physicians to use together when deciding on treatment plans [42]. Although this framework is limited in its treatment options, it provides an example of cooperative decision making, which has the potential to provide NET patients with increased autonomy and feelings of improved communication with their physician.

In summary, there is a lack of NET-specific tools that have the goal of increasing patients’ self-knowledge of their diagnosis and treatment journey.

## 3. NET VITALS

**Figure 1:**
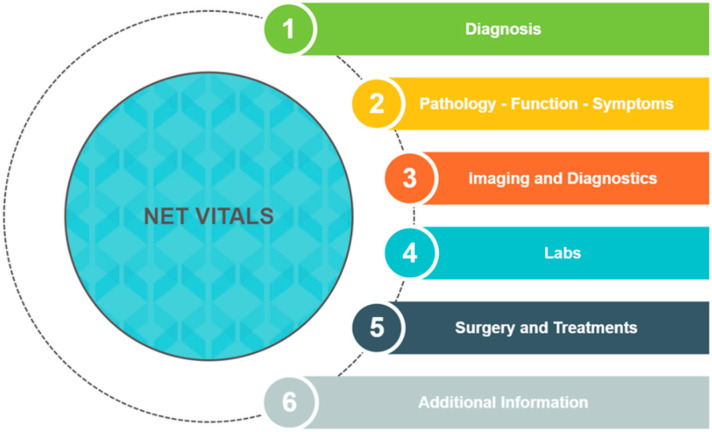
Sections of NET VITALS.

In light of complaints from NET patients that they did not have enough information about their diagnosis and treatment from their physician [7,8,9], we decided to create a tool to allow patients to address these issues. Patient advocates from the Learn Advocate Connect Neuroendocrine Tumor Society (LACNETS) collaborated with NET physicians from City of Hope to create NET VITALS, a patient-centered self-assessment tool [43,44]. NET VITALS comprises six sections: diagnosis information, pathology/functional status/symptoms, imaging and diagnostic information, laboratory test results, surgery and treatments received, and additional information (genetic testing information, level of social support) (Figure 1 and Figure S1) [43]. The goal of NET VITALS is to give patients a sense of autonomy and control as they navigate their NET diagnosis and treatment odyssey.

In 2019, we introduced NET VITALS to patients attending the Los Angeles NET Education Conference [43]. Patients were invited to complete NET VITALS after attending a seminar that explained how to fill it out. The feasibility of NET VITALS was demonstrated, with an 88.3% response rate (68 out of 77 patients) and a median of 85.7% of items completed. NET patients were satisfied with NET VITALS as a potential tool to use with their physicians, with 74.6% of patients agreeing that NET VITALS was a useful communication tool and 76.3% of patients indicating that they would recommend NET VITALS to someone else. In terms of disease and treatment knowledge, NET VITALS highlighted areas where NET patients may not have as much knowledge about their diagnosis or treatment, including tumor Ki-67 index, grade, functional status, differentiation status, and receipt of liver-directed therapy. These gaps in knowledge suggest that NET VITALS could be used to spur communication between patients and physicians to increase NET patients’ knowledge of their disease.

Given the feasibility and high level of patient satisfaction with NET VITALS in our preliminary cross-sectional survey study, we present a suggested infrastructure for implementing NET VITALS in the clinic.

## 4. Implementation of NET VITALS: The City of Hope Model

**Figure 2:**
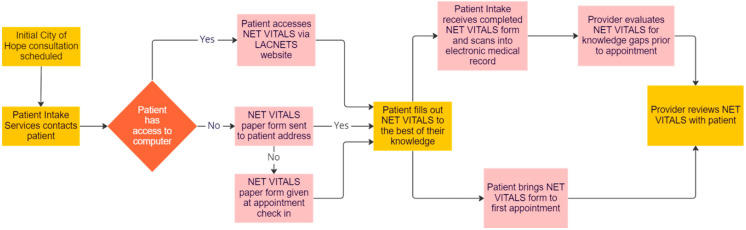
Proposed implementation of NET VITALS.

NET patient advocates, physicians experienced in treating patients with NETs across the City of Hope enterprise, and healthcare providers with experience integrating patient assessments into clinical care were approached to determine the best way to implement NET VITALS into clinical practice. A proposed model for NET VITALS integration is shown in Figure 2.

In this model, NET VITALS will be built into the list of intake tasks for patients with NETs seeking consultation at City of Hope. Intake coordinators will direct patients to the LACNETS website to download and complete NET VITALS. Once completed, the patient will send a copy of NET VITALS to the intake coordinator to scan and upload into the patient’s medical record for physician review prior to the consultation appointment.

For patients unable to access the LACNETS website, the intake coordinator will mail a paper copy of NET VITALS to the patient for completion prior to their appointment. If completion of NET VITALS prior to the consultation appointment is not feasible, patients will be given a paper copy of NET VITALS upon check-in on the day of their appointment. Paper copies of NET VITALS will be scanned and uploaded into the patient’s medical record on the day of their initial consultation at City of Hope.

## 5. Opportunities and Challenges of NET VITALS Integration within the City of Hope Enterprise

The implementation model for NET VITALS will be piloted initially on the City of Hope main Duarte campus and select community practice sites throughout southern California. NET VITALS will also continue to be promoted through the LACNETS website and outreach platform. Once the feasibility of this model has been demonstrated, expansion to all City of Hope locations across the enterprise will be performed.

Strengths of the proposed infrastructure include the strong relationships between the academic and community centers of City of Hope. With a primary center in Duarte, the City of Hope Orange County Lennar Foundation Cancer Center in Irvine, over two dozen community practice sites across southern California, and three Cancer Treatment Centers of America locations in Phoenix, Chicago, and Atlanta, the potential for collaboration is enormous. The connectivity of the main campus at Duarte and various community practice locations has been previously demonstrated [45]. This interconnectivity allows physicians from satellite clinics that may not see many patients with NETs on a routine basis to have access to specialists at other locations to better understand their patient’s diagnosis and develop an optimal treatment plan. City of Hope’s new relationship with Cancer Treatment Centers of America furthers this connectivity, allowing patients at all locations to have the opportunity to potentially benefit from NET VITALS.

A potential challenge of this implementation strategy is its reliance on electronic medical records. To complete NET VITALS before their appointment, patients are expected to have access to their online patient portal and the LACNETS website (which also implies Internet access). We have suggested alternative pathways to complete NET VITALS that are not dependent on Internet access to ensure that all patients have an opportunity to complete this assessment. Additionally, City of Hope is in the process of promoting access to the online patient portal, which includes converting Cancer Treatment Centers of America to the electronic medical record system used by City of Hope to ensure uniform access across the enterprise. The integration of NET VITALS into the electronic medical record could allow care teams to easily compare patients’ responses to pre-existing data and track patients’ care over time.

Another perceived limitation may be the inability of patients to complete all sections of NET VITALS before their appointment. However, patients are not expected to be familiar with everything covered in NET VITALS [44]. Identifying areas where knowledge is lacking allows patients to have a more guided discussion with their physician during their appointment, potentially improving overall patient–physician communication.

## 6. Conclusions

Patient-centered self-assessments, such as NET VITALS, may help increase patients’ knowledge about their NET diagnosis/treatment and promote dialogue with their physician and healthcare providers. Identifying and implementing a strategy for the incorporation of NET VITALS into clinical care can be significant to care teams that strive to provide the best personalized care possible for patients with NETs.

## Data Availability

Not applicable.

## Supplementary Materials

The following supporting information can be downloaded at: https://www.mdpi.com/article/10.3390/jcm12031229/s1, Figure S1: NET VITALS.
