# Supplementary material for: Proposed Implementation of a Patient-Centered Self-Assessment Tool for Patients with Neuroendocrine Tumors among Academic and Community Practice Sites: The City of Hope Model
Source: J Clin Med. 2023 Feb 3;12(3):1229. doi: 10.3390/jcm12031229 (PMC9917881; doi:10.3390/jcm12031229)
Supplement: Supplementary file 1 [file jcm-12-01229-s001.zip › jcm-2133161-supplementary.pdf]

General Information

Today's date

Patient name
last, first

Gender

- [ ] Female
- [ ] Male

Date of birth
mm/dd/yy

Year of diagnosis
mm/dd/yy

Phone number

Email

1. My neuroendocrine tumor (NET) was found:
check all that apply

- [ ] Incidentally (routine exam, unrelated procedure)
- [ ] Due to obstruction
- [ ] NET symptoms/carcinoid syndrome
- [ ] *Other*

Part 1 | Diagnosis

2. I am a neuroendocrine tumor (NET) patient and my primary (origin) tumor is:

- [ ] Thymus
- [ ] Small intestine (ileum)
- [ ] Rectum
- [ ] Stomach
- [ ] Proximal colon
- [ ] Lung
- [ ] Appendix
- [ ] Pheo/para
- [ ] Pancreas
- [ ] Distal colon
- [ ] Unknown
- [ ] *Other*

3. My NET has spread (metastasized) to *(check all that apply)*:

- [ ] Not metastatic at this time

LOCATION	AT DIAGNOSIS	PROGRESSION/NEW IMAGING	DATE *mm/dd/yy*
[ ] Liver			
[ ] Bones			
[ ] Mesentery			
[ ] Peritoneum			
[ ] *Other*			

Part 2 | Pathology - Function - Symptoms

4. My Ki-67 index is ______ % found on Pathology Report dated ______ .

- [ ] I brought my Pathology Report

Changed from previous biopsy?

- [ ] Yes
- [ ] No
- [ ] N/A

5. My neuroendocrine tumor is *(select an answer in each row)*:

ROW 1	[ ] Well-differentiated	[ ] Poorly differentiated	[ ] Don't know	
ROW 2	[ ] **Grade 1** Mitotic index <2, Ki-67 0-2	[ ] **Grade 2** Mitotic index 2-10, Ki-67 3-20	[ ] **Grade 3** Mitotic index >10, Ki-67 >20	[ ] Don't know

This document is intended for informational and educational purposes only and is not a substitute for professional medical advice. Always consult with your physician or health care provider. © LACNETS

Part 2 | Pathology - Function - Symptoms - cont'd

6. My neuroendocrine tumor is *(indicate most current)*:

☐ Functional (hormone-producing) ☐ Non-functional ☐ Don't know

7. I have symptoms *(check all that apply)*:

☐ Diarrhea ☐ Weight loss ☐ Fatigue ☐ Abdominal cramping ☐ Palpitations/fast heartbeat

☐ Wheezing ☐ Flushing ☐ Pain ☐ Shortness of breath ☐ Hypertension/high blood pressure

☐ Bloating/gas ☐ *Other* ______

8. I have had symptoms for ____ years *(if applicable)*.

Part 3 | Imaging & Diagnostics

9. I have had the following types of imaging, and possess the disk and/or report *(if applicable)*:

TYPE OF IMAGING	DATE(S) / DATE RANGE			DISK	REPORT
☐ MRI (3 most recent)		–		☐	☐
☐ CT (3 most recent)		–		☐	☐
☐ Octreotide scan		–		☐	☐
☐ DOTATATE PET (i.e. Ga68 PET/CT or Cu64 PET/CT)		–		☐	☐
☐ FDG-PET		–		☐	☐
☐ PET		–		☐	☐
☐ Echocardiogram		–		☐	☐
☐ Colonoscopy		–		☐	☐
☐ Upper Endoscopy		–		☐	☐
☐ MIBG		–		☐	☐
☐ Capsule Endoscopy		–		☐	☐
☐ *Other* ______		–		☐	☐
☐ *Other* ______		–		☐	☐

This document is intended for informational and educational purposes only and is not a substitute for professional medical advice. Always consult with your physician or health care provider. © LACNETS

Part 4 | Labs

10. I have had the following labs and possess the report *(if applicable)*. Indicate if abnormal.

TYPE OF LAB	DATE(S) / DATE RANGE		REPORT
☐ Complete Blood Count (CBC)		–	☐
☐ Basic Metabolic Panel (BMP) or Complete Metabolic Panel (CMP)		–	☐
☐ Chromogranin A (CgA)		–	☐
☐ Serotonin		–	☐
5-HIAA ☐ urine ☐ plasma		–	☐
☐ Plasma (blood) Metanephrine and/or 24-Hour Urine		–	☐
☐ Pancreastatin		–	☐
☐ Neurokinin A		–	☐
☐ A1C		–	☐
☐ *Other*		–	☐
☐ *Other*		–	☐
☐ *Other*		–	☐

Part 5 | Surgery & Treatments

TYPE	DATE(S)	LOCATION	PHYSICIAN	REPORT
**11. Surgery to remove primary tumor(s) *(Indicate all that apply)***				
☐ Small bowel				☐
☐ Pancreas				☐
☐ Lung				☐
☐ Adrenal				☐
☐ *Other*				☐
**12. Surgery to remove metastatic tumor(s) *(Indicate all that apply)***				
☐ Liver				☐
☐ Spleen				☐
☐ Lung				☐
☐ *Other*				☐

This document is intended for informational and educational purposes only and is not a substitute for professional medical advice. Always consult with your physician or health care provider. © LACNETS

TYPE	DATE(S)	LOCATION	PHYSICIAN	REPORT
**13. Other surgery**				
☐ Gallbladder removal (Cholecystectomy)				☐
☐ *Other*				☐
☐ *Other*				☐
**14. Liver-directed treatment** *(indicate all that apply)*				
☐ Radiofrequency ablation				☐
☐ Chemoembolization (TACE)				☐
☐ Bland embolization				☐
☐ Radioembolization (Thera-Sphere®,SIR-Spheres®,TARE)				☐
☐ *Other*				☐

15. Somatostatin Analogs (SSA) & others *(indicate all that apply)*

TYPE	DOSE	FREQUENCY	START DATE	LAST DOSE (DATE)
☐ Sandostatin LAR (octreotide, long-acting)				
☐ Sandostatin (octreotide, short-acting)				
☐ Somatuline Depot (lanreotide)				
☐ Xermelo (telotristat etiprate)				

16. Chemotherapy / Biologically-Targeted / Immunotherapy, including Clinical Trials/ PRRT (Peptide Receptor Radionuclide Therapy, i.e. Lutathera®) *(indicate all that apply)*

TYPE	DOSE/FREQUENCY	START DATE	END DATE	PHYSICIAN
☐ Xeloda (capecitabine)		–		
☐ Temodar (temozolomide)		–		
☐ CAPTEM (capecitabine & temozolomide)		–		
☐ Afinitor (everolimus)		–		
☐ Sutent (sunitinib)		–		
☐ Cisplatin/etoposide		–		
☐ PRRT		–		
☐ *Other*		–		
☐ *Other*		–		

This document is intended for informational and educational purposes only and is not a substitute for professional medical advice.
Always consult with your physician or health care provider. © LACNETS

Part 6 | Additional information

17. I am diabetic:

☐ Type I ☐ Pre-diabetic ☐ Type II ☐ I am not diabetic

Notes on diabetes (including diet-controlled, taking meds, taking insulin, physician managing diabetes):

18. Allergies *(medications/food)*:

19. Family history *(specify cancer and/or rare diseases)*:

20. I have had Genetic Testing:

☐ Yes ☐ No ☐ Don't know ☐ Mutation(s) found

21. Medications/supplements *(including vitamins, digestive enzymes)*:

22. Current physician(s) & medical team *(Including names, institution, phone numbers)*

Primary care provider:

Medical oncologist:

Surgeon:

Nuclear medicine physician:

Other providers *(i.e. endocrinologist, cardiologist, navigator)*:

23. Social support

Number in household:

Employment Status: ☐ Full-time ☐ Part-time ☐ Retired ☐ On disability

Has employment been affected by diagnosis?

This document is intended for informational and educational purposes only and is not a substitute for professional medical advice.
Always consult with your physician or health care provider. © LACNETS

Notes to ask my physician:

Resources (available at www.LACNETS.org/resources)

More resources at www.LACNETS.org/resources

LACNETS - Learn Advocate Connect Neuroendocrine Tumor Society | www.LACNETS.org

Canadian NET patient resource guide, definitions, reference guide | bit.ly/CNETS_Resource

Cancer Legal Resources Center: legal, disability, insurance, financial | www.cancerlegalresources.org

Carcinoid Cancer Foundation - Checklist for the newly diagnosed | www.carcinoid.org

Healing NET Foundation (see NET PRIMER PDF) | www.thehealingnet.org

International Neuroendocrine Cancer Alliance | http://incalliance.org

NET glossary | www.knowyournets.com/materials

Neuroendocrine Tumor Research Foundation | www.netrf.org

North American Neuroendocrine Tumor Society | https://nanets.net/resources/patient-resources

Northern California CarciNET Community | www.NorCalCarciNET.org

PRRT Info (by patient advocates Josh Mailman & Gary Murfin) | www.PRRTinfo.org

Triage Cancer - Resource for legal, insurance, financial | www.triagecancer.org

This document is intended for informational and educational purposes only and is not a substitute for professional medical advice.
Always consult with your physician or health care provider. © LACNETS
